# Method of Detecting the Presence of Cotton in Linen

**Published:** 1847-10

**Authors:** 


10. *Method of detecting the presence of Cotton in Linen.*—M. Kindt, a Bohemian apothecary proposes to detect the presence of cotton in linen, by a process based upon the principal that the fibre of cotton is more rapidly dissolved in concentrated sulphuric acid than that of either hemp or flax. The cloth having been thoroughly deprived of its dressing by being boiled some time in water, should be well dried. One end of the piece should then be plunged in concentrated sulphuric acid, and left in it from one to two minutes. The cloth becomes transparent, and should be well washed in water, rubbing it with the fingers, if necessary, to favor the removal of the gummy matter which has been produced. It should now be rinsed in water holding in solution a small quantity of potash or other alkaline substance to neutralize any acid it may still contain, and again in pure water, and finally dried. If the cloth contains any cotton this will have been dissolved, and its absence may be readily detected by comparing the portion subjected to the acid with that which was not.

If the specimen were allowed to remain too long in the sulphuric acid, the linen fibres would be acted upon, but if the cloth were made entirely of flax the corosion would be uniform. The cotton however is always first acted upon, and is converted into gum whilst the linen threads still remain white and opaque.—Translated from Journ. de Pharm., 1847.—Bull. Gen. Therap., for *Ibid.*

